# Toward the Development of tES- Based Telemedicine System: Insights From the Digital Transformation and Neurophysiological Evidence

**DOI:** 10.3389/fpsyt.2022.782144

**Published:** 2022-07-11

**Authors:** Takashi Ikeda, Keiichiro Nishida, Masafumi Yoshimura, Ryouhei Ishii, Banri Tsukuda, Tomoyasu Bunai, Yasuomi Ouchi, Mitsuru Kikuchi

**Affiliations:** ^1^Research Center for Child Mental Development, Kanazawa University, Kanazawa, Japan; ^2^United Graduate School of Child Development, Osaka University, Osaka, Japan; ^3^Department of Neuropsychiatry, Kansai Medical University, Osaka, Japan; ^4^Department of Occupational Therapy, Faculty of Rehabilitation Kansai Medical University, Osaka, Japan; ^5^Department of Neuropsychiatry, Kansai Medical University Medical Center, Osaka, Japan; ^6^Occupational Therapy Major, Graduate School of Rehabilitation Science, Osaka Metropolitan University, Habikino, Japan; ^7^Department of Biofunctional Imaging, Preeminent Medical Photonics Education & Research Center, Hamamatsu University School of Medicine, Hamamatsu, Japan; ^8^Department of Psychiatry and Neurobiology, Graduate School of Medical Science, Kanazawa University, Kanazawa, Japan

**Keywords:** digital transformation (DX), telemedicine, transcranial direct current stimulation (tDCS), home-use device, cognitive function, emotion regulation, mood disorders, electroencephalography (EEG)

## Introduction

The coronavirus pandemic had considerable effects on people's emotions, such as fear of infection, anxiety, and depressive mood (1, 2). From the perspective of preventing the spread of the virus, it would be ideal for everyone to avoid all occasions of interpersonal contact. To solve this problem, there may be a need to establish a telemedical system for the treatment of mental diseases (3).

At present, there is an increasing use of online diagnosis with digital transformation (DX) technologies, typically via telemedicine (4, 5). DX involves using information technology to transform existing mechanisms in order to provide new services (6). One advantage of medical care using DX is the ability to take action anywhere and anytime, which reduces labor costs and other expenses for both patients and healthcare providers.

Recent advancements in psychiatric treatment include non-invasive brain stimulation (NIBS) techniques, such as transcranial magnetic stimulation and transcranial electrical stimulation (tES) (7, 8). Several studies have examined the efficacy of NIBS techniques in treating psychiatric diseases (9). For example, transcranial direct current stimulation (tDCS) is a NIBS technique wherein simple equipment is used to pass a weak direct current between electrodes placed on the scalp. Due to the high level of safety with tDCS (10), there has been a sharp increase in high-quality research involving tDCS on cognition and emotion regulation (11, 12), and treatment with tDCS devices is considered suitable for use in telemedicine. It is ideal for home-based medical care because treatment is provided entirely by electronic devices, and it is easy to manage changes in stimulation parameters (13). Consequently, several ideas for telemedicine using tDCS have been proposed (14–23). Recently, a telemedicine depression trial was conducted with tDCS (24). Given the popularity of the research and the need for more telemedicine options highlighted by the COVID-19 pandemic, we sought to discuss the future of tDCS-telemedicine systems compatible with DX.

## Overview of Neurophysiology

Coronavirus infections not only threaten human life but also leave serious sequelae after recovery. Several reports have shown the significant decrease of cognitive function in groups of former patients who had apparently recovered from infection (25, 26), signifying negative effects on the working memory (WM) system that supports higher cognitive function (27). These dysfunctions are governed by the prefrontal cortex (28, 29). When studying brain interventions, it is important to concurrently evaluate how tDCS stimulation changes brain activity.

For future applications of telemedicine at home, electroencephalography (EEG) is likely to be the leading neuroimaging technique that can be used. Thus, we provide an overview of studies that targeted the dorsolateral prefrontal cortex (DLPFC) and investigated the effects of tDCS via EEG and magnetoencephalography (MEG), which can offer practically equivalent indices to EEG.

### Cognitive Function

Numerous studies have examined DLPFC stimulation for improving cognitive function, but the results are mixed. While some reports have indicated significant improvement effects, others have not shown any effects or indicated reduced cognitive performance (30–32). However, even when no behavioral changes were observed, it is possible that brain activity patterns had changed. Therefore, researchers need a process using neurophysiological evidence to verify whether these changes have triggered the expected behavior and, thereby, determine the efficacy of tDCS treatment.

Regarding brain activity, which is closely related to cognitive functions measured by EEG, oscillation analysis can extract the activity within a specific frequency bandwidth (33, 34). The effect of tDCS depends on the state of the participant (35). For example, research comparing patients with mild cognitive impairment and healthy participants suggests that the effects of tDCS for elevating cognitive functions cannot be measured in fully functioning participants (36). Conversely, tDCS performed with four patients who had experienced brain damage resulted in improved WM, raising our hopes for its application in the rehabilitation sector (37).

One meta-analysis targeted patients with a disease but did not evaluate their brain function. They reported that, although tDCS was shown to improve mood disorders in some patients and cognitive function in patients with schizophrenia, these results were not seen in patients with Alzheimer's dementia or MCI (38). Another meta-analysis showed that tDCS promoted cognitive function in patients with schizophrenia (39). Regarding the lack of effects of elevated cognitive function in a meta-analysis of tDCS targeting healthy participants (40), it is possible that the effects of tDCS differ between healthy participants and patients with mood disorders (41). Within the population of patients whose cognitive function has temporarily declined, a group exists in which tDCS might be effective.

### Mood

Through our overview, we found that the analytical methods used when simultaneously evaluating the effects of tDCS on emotions and neurophysiology are rich in variety. When the cathode was placed above the left DLPFC and the anode was placed on the contralateral supraorbital area, the subjective magnitude of pain measured by the visual analog scale slightly decreased by active stimulation, despite no changes observed in visually inspected EEG (42). Maeoka et al. (43) examined whether pain-related unpleasant emotions induced by unpleasant photos were decreased by tDCS. A decrease in alpha power and an increase in beta power were observed after stimulation in the active-stimulation group.

In another study, researchers attempting to stimulate the ventromedial prefrontal cortex placed cathodes underneath the chin of participants and asked them to look at some happy faces and some fearful faces. According to their results, happy faces were associated with greater neural reactivity arousal (44). When the same regions were stimulated with tDCS, looking at pleasant photographs increased the strength of the magnetic field presumed to be generated by the medial prefrontal cortex (45).

Palm et al. (46) examined a patient with depression and evaluated the effects of tDCS with anodes placed above the left DLPFC, employing a quantitative EEG analysis method called low-resolution brain electromagnetic tomography (LORETA) (47). The results showed a decrease in current densities in the frontal region. LORETA has examined the predicted changes in anxiety owing to tDCS and described its possibilities (48). The use of EEGs also has diverse hidden possibilities. Additionally, the components of event-related potential N170, P3, and N400 are being investigated (49–51). Given all of this new evidence and the continuing progress in methods of analysis, we anticipate further growth and development in the field.

### Neurophysiological Indices

**Figure 1:**
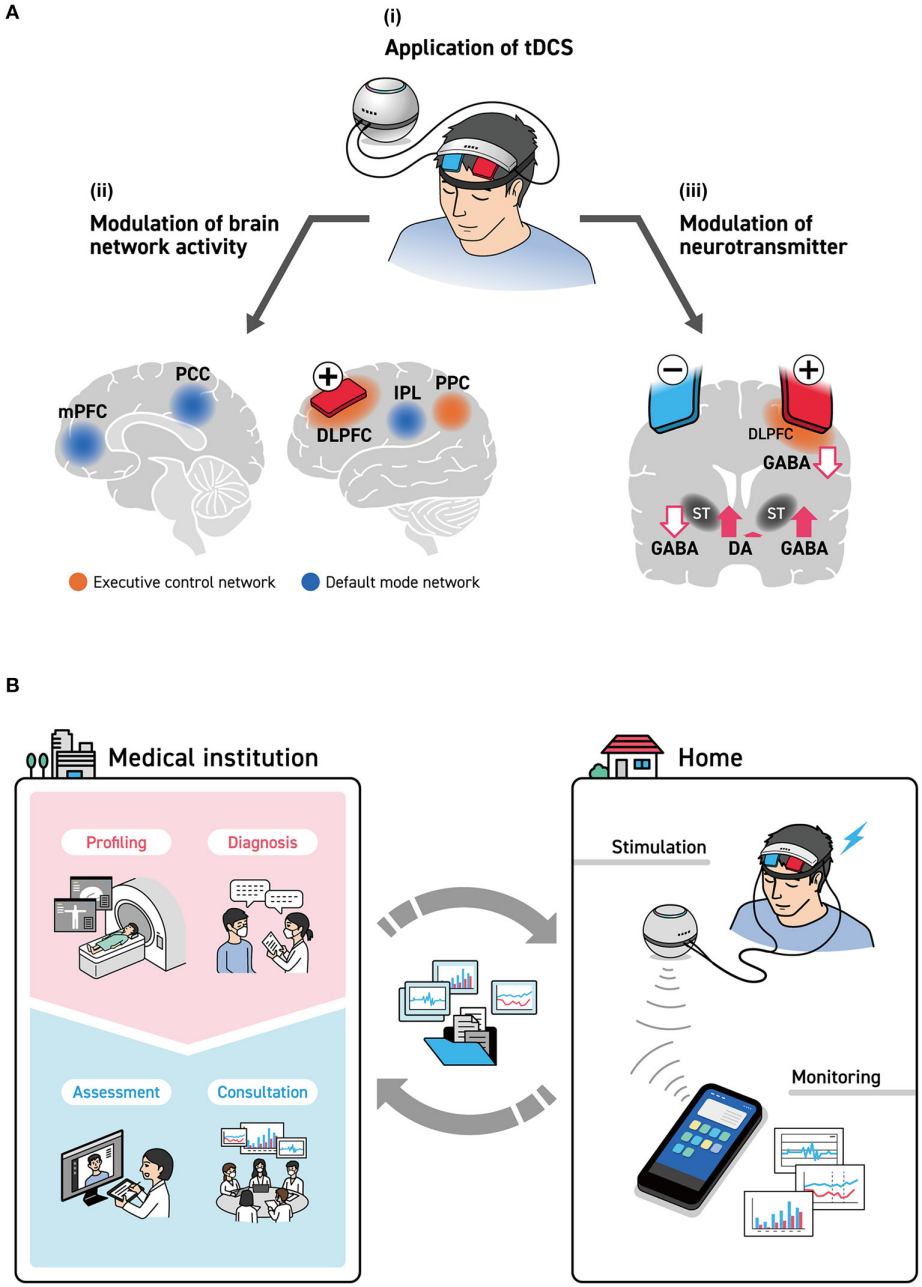
**(A)** brain stimulation by tDCS. (i) With the intent of stimulating the left DLPFC to improve cognitive function and mood, an anode was installed on F3 as defined under the International Standard ten-twenty electrode system. Many studies, however, have installed cathodes on either the F4 of the right hemisphere or the forehead. (ii) The spread of the effects of tDCS to the cerebral cortex, as estimated by the EEG/MEG studies are seen with the executive control network (DLPFC, SFC, and PPC) and the default mode network (mPFC, PCC, and IPL). (iii) The influence of tDCS stimulation of the left DLPFC on neurotransmitters. This brings about the release of dopamine in the right striatum and a reduction in GABA concentration, while it brings about an increase in GABA concentration in the left striatum. DLPFC, dorsolateral prefrontal cortex; IPL, inferior parietal lobule; mPFC, medial prefrontal cortex; PCC, posterior cingulate cortex; PPC, posterior parietal cortex; SFC, superior frontal cortex; ST, striatum. **(B)** tDCS-based telemedicine system, including medical institutions. In hospitals, meticulous examinations and brain measurements are done by physicians and specialists, who formulate stimulation parameters. Here, the structural data of the head were obtained by CT and MRI, and more detailed data on brain functions were obtained by fMRI, MEG, and PET to determine the optimal positions on the scalp for providing tDCS as well as parameters that are suited to individual patients. Brain condition before and after stimulation was measured by simple EEG, and the effects of tDCS were monitored as needed. These data are delivered to medical specialists via smart devices and are used to confirm the effects of treatment and formulate the next treatment plan.

Various neurophysiological indices are being used to track cognition and emotion, as evaluations based on neurophysiological indices using only stimulation sites are insufficient. To evaluate the state of the brain of each participant, it is necessary to consider what is happening inside each brain region. This is because, as a theory suggests, there are possible differences between healthy individuals and patients with depression in terms of the sites and balance of brain activation (52). Understanding the neurophysiological basis of tDCS on neural oscillations in the local activation and inter-regional neural networks and neurotransmitters will be the key to evaluating the state of the individual brain (Figure 1A).

The influence of NIBS on the brain function network has been previously reported (53). For example, stimulation applied to the DLPFC affects not only the executive control network (ECN), which is important for the WM, but also the default mode network (DMN), which is inversely correlated with ECN (54). An earlier study on the simultaneous measurement of fMRI and EEG found an inverse correlation with EEG activity between the frontoparietal network, which is a type of ECN, and the alpha band (55). Other studies focused on the relationship between DMN and EEG activity in the alpha and beta bands, as well as on the relationship between DMN and a combination of alpha and theta bands (56). The relationship between brain stimulation and RSNs is likely to attract growing research attention.

The effects of tDCS are often explained on the basis of electrophysiological mechanisms such as induced depolarization and hyperpolarization of the membrane potential (57). However, more studies have looked at the manifestation of its effects from a different perspective, such as those that support the remote effect hypothesis of tDCS, claiming that it affects the neurotransmitter system in the deep brain. In a study related to dopamine using PET, researchers performed tDCS by placing anodes on the left DLPFC and cathodes on the right DLPFC; they found that dopamine was released from the right ventral striatum and that attention and execution functions were reinforced (58). The effect of tDCS on the GABA system, which plays an important role in the modulation of the basal ganglia, was shown in a study using magnetic resonance spectroscopy (59). Here, GABA levels had decreased, unlike the release of dopamine from the right striatum seen with tDCS, and GABA concentration had conversely increased in the left striatum. tDCS appears to have an effect not only on the brain's surfaces but also on the neurotransmitter systems in the basal ganglia found in the deep brain.

## Discussion

Using DX technology may make it possible to integrate the necessary information into smart devices to create efficient systems to provide mental healthcare that minimizes the burden on both the medical institution and the patient. A technique that is suited for evaluating whether stimulation has reached the patient's brain is EEG, which, in addition to being safe and convenient, has a long-known history related to numerous neuromarkers. Also, as wireless battery-operated EEG devices are already commercially available, it should not be too difficult to produce integrated devices.

### tDCS-Based Telemedicine System Including Medical Institutions

Researchers have noted individual differences in the state of the brain, including functional and biological changes caused by the presence of disease (60), and differences in the histological structures of the brain, including the skull (61). It may be considered that tDCS is moving in the direction of individual optimization, as seen in recent trends in depression treatment (62). Herein, we clarified the roles of tDCS in telemedicine, especially for home-based care; simultaneously, we discussed the need to build an integrated system that will allow specialists at medical institutions to verify its effects (Figure 1B).

The key here is to create a system that enables profiling and diagnoses at medical institutions rather than requiring participants at home to complete the process on their own. The emergence of the effects of treatment appears to be influenced by the difference in the activity of existing functional networks and neurotransmitters. Therefore, measures to obtain detailed structural images of the head via MRI, ascertain the activity status via brain function imaging methods (63), and enhance the provision of effect predictions will probably be effective in the near future. The efficacy of tDCS will be enhanced by using these predictions as a starting point and then optimizing various parameters, such as the positions of the tDCS electrodes, electrical current levels, and stimulation time.

Information on the situation and effects should be communicated to the hospital via mobile devices. Although the electrical current used in tDCS is weak, cases of adverse reactions—such as burns and skin discomfort—have been reported (64). However, these problems can be resolved along with the further development of the equipment with monitoring. The information such as on/off-line effect and with/without stress is also important (65, 66).

Digital technologies must be used to send large data safety. Although this capacity may not be achievable until the equipment and Internet device technologies are more evolved, these goals could be reached in the near future, given the rate of innovation in the Internet of Things.

The relationship between the social system and industry is important to the future development of tDCS usage. It is necessary to prepare the social infrastructure, including the human resources necessary to run the medical system and develop training (24, 67). Quality control of devices is essential to ensure the safety of users in case of failure, and technology must be developed to enhance data transmission security (68).

After implementation, the day's stimulation status and the brain activity data are sent to medical institutions for assessment; then, based on the data, therapeutic policies are decided following consultation within a team. Current video conferencing systems allow physicians and patients to communicate whenever necessary, and physicians can check on a patient's current status, discuss consultations, and adjust stimulation parameters without the patient having to visit a medical institution. However, if treatment policies were determined by multiple specialists rather than the personal judgment of one individual, be it a patient or healthcare provider, treatment appropriateness and safety would be better guaranteed.

## Conclusions

To guarantee safety and efficacy, researchers must make gains in technology, uniting tES and neurophysiology. Additionally, the establishment of a system that allows for medical institutions to provide comprehensive evaluations and consultations with expert follow-ups is vital for tES used in telemedicine. This concept cannot be realized by the healthcare industry alone, and cooperation with industries and social systems will be necessary. Now that awareness of the productivity and importance of telemedicine has grown because of the coronavirus pandemic, these perspectives toward the future have become realistic and comprise the visions for the future that we can pursue.

## Author Contributions

TI and KN conceptualized and wrote the draft. TI, KN, and YO reviewed the work. All authors contributed to the article and approved the submitted version.

## Funding

All authors received funding from the Osaka University Centre of Innovation (COI) program of the Japan Science and Technology Agency (JST, No. JPMJCE1310). KN received Grant-in-Aid for Young Scientists (B) (KAKEN 26860950) and MY received a Grant-in-Aid for Scientific Research (C) (KAKEN 19K08056). This work was partially supported by The Collaborative Research Network for Asian Children with Developmental Disorders (CRNACDD).

## Conflict of Interest

The authors declare that the research was conducted in the absence of any commercial or financial relationships that could be construed as a potential conflict of interest.

## Publisher's Note

All claims expressed in this article are solely those of the authors and do not necessarily represent those of their affiliated organizations, or those of the publisher, the editors and the reviewers. Any product that may be evaluated in this article, or claim that may be made by its manufacturer, is not guaranteed or endorsed by the publisher.
